# Editorial: Recent advances in research on cognitive frailty and related conditions

**DOI:** 10.3389/fnagi.2025.1595526

**Published:** 2025-04-07

**Authors:** Takao Yamasaki, Mutsuhide Tanaka, Shuzo Kumagai

**Affiliations:** ^1^Department of Neurology, Minkodo Minohara Hospital, Fukuoka, Japan; ^2^Kumagai Institute of Health Policy, Fukuoka, Japan; ^3^Department of Health and Welfare Occupational Therapy Course, Faculty of Health and Welfare, Prefectural University of Hiroshima, Hiroshima, Japan

**Keywords:** cognitive frailty, subjective cognitive decline, mild cognitive impairment, motoric cognitive risk syndrome, sarcopenia, risk factor, intervention, biomarker

## 1 Introduction

Cognitive frailty (CF) is an age-related condition that combines physical frailty and cognitive impairment without dementia (Kelaiditi et al., 2013). Conversely, physical frailty includes unintentional weight loss, exhaustion, weakness, slow walking speed, and low physical activity (Fried et al., 2001). This dual vulnerability significantly increases the risk of dementia, functional disability, reduced quality of life, hospitalization, and mortality (Chen et al., 2022).

Frailty encompasses other phenotypes, such as social and oral frailty. Social frailty refers to declining social resources critical for basic human needs (Bunt et al., 2017), whereas oral frailty involves the deterioration of oral functions among older adults (Tanaka et al., 2018). Moreover, both increase the risk of dementia (Choi and Ko, 2024; Nagatani et al., 2023).

Other dementia-related high-risk states include subjective cognitive decline (SCD), mild cognitive impairment (MCI), motoric cognitive risk syndrome (MCR), and sarcopenia (Yamasaki and Ikeda, 2024). SCD is characterized as a self-perceived cognitive decline without objective deficits (Jessen et al., 2020), whereas MCI involves measurable deficits but intact daily functioning (Alzheimer's Association., 2024). Both progress along the Alzheimer's disease continuum (Yamasaki and Ikeda, 2024).

MCR is another pre-dementia condition characterized by subjective cognitive complaints and slow gait (Verghese et al., 2014). Sarcopenia, which is defined as age-related skeletal muscle loss and diminished function, is recognized as a risk factor for both cognitive decline and dementia (Cruz-Jentoft et al., 2019; Amini et al., 2024).

Since these conditions are partially reversible, early risk identification and interventions could delay adverse outcomes. Therefore, the aim of this Research Topic is to collect research on advances in understanding high-risk dementia states, focusing on risk factors, innovative interventions, and biomarkers.

## 2 New insights into risk factors for cognitive decline and its prevention

Dementia risk is significantly influenced by 14 modifiable factors, including low education, traumatic brain injury, physical inactivity, smoking, excessive alcohol consumption, hypertension, obesity, diabetes, hearing loss, depression, social isolation, air pollution, poor vision, and high low-density lipoprotein cholesterol. Addressing these factors could prevent up to 45% of dementia cases globally (Livingston et al., 2024). This Research Topic encompasses five studies evaluating the risk factors contributing to high-risk states of dementia, focusing on 14 identified factors as well as additional potential risks.

By leveraging group-based dual trajectory modeling, Ji et al. identified a subgroup of older adults characterized by poor cognitive function and higher levels of frailty. This group was significantly associated with several factors, including being female, older age, low levels of education, residing in rural areas, being unmarried, and having comorbidities such as hypertension, diabetes, complete tooth loss, vision impairment, and hearing impairment.

Dong Q. et al. conducted a latent profile analysis to categorize community-dwelling older adults based on cognitive function, physical frailty, and social frailty, revealing two distinct subgroups: one characterized by high cognitive function and low frailty, while the other by low cognitive function and high frailty. Individuals in the latter group were predominantly aged 80 years, had lower income levels, faced multiple chronic conditions, and exhibited moderate to poor health status.

Qin et al. suggested that age, lower educational attainment, malnutrition, and depression are significant risk factors for CF. Moreover, their findings emphasized a significant correlation between mitochondrial dysfunction and CF, suggesting that mitochondrial dysfunction in the peripheral blood could serve as a potential phenotype of CF.

Dong X. et al. also investigated the relationship between sarcopenia and cognitive function, with a particular focus on the cognitive subdomains impacted by sarcopenia. Their findings revealed a clear correlation, as individuals with sarcopenia exhibited diminished performance in overall cognitive function as well as specific subdomains. Of note, cognitive abilities such as fluency declined progressively with the increasing severity of sarcopenia.

In a community-based prospective cohort study, Li S. et al. revealed a compelling connection between baseline cognitive decline and all-cause mortality among individuals aged 60 years. Their findings emphasized that both mild and moderate-to-severe cognitive impairments, as well as rapid cognitive decline, significantly increased the risk of mortality from all causes.

By synthesizing the findings of these five studies, two key strategies have been established. First, preventing high-risk states for dementia requires regular health monitoring and timely intervention to address modifiable risk factors. Second, the early detection of cognitive decline through consistent cognitive assessments is essential. Moreover, targeted interventions can then be used to delay cognitive impairment progression and mitigate the associated mortality risks.

## 3 Innovative approaches for preventing cognitive decline

Preventive strategies for cognitive impairment include both pharmacological and non-pharmacological therapies. Non-pharmacological approaches include cognitive training, physical exercise, dietary interventions, art-based therapy, reminiscence therapy, and aromatherapy (Maneemai et al., 2024). This Research Topic also emphasized three promising preventive interventions.

For research on pharmacological therapy, Wang and Li investigated a cohort of community-dwelling individuals with dyslipidemia and found that sustained statin use was associated with better cognitive outcomes compared with non-use. The benefits were particularly pronounced in individuals aged 65 years, suggesting that statins could play a protective role in preserving cognitive function in the elderly.

Jhan et al. investigated the effects of light-intensity physical activity on cognitive function among community-dwelling older adults as part of their research on non-pharmacological therapy. Their findings suggested that engaging in at least 3 h of light-intensity physical activity per day plays a crucial role in preserving and enhancing orientation-related cognitive function over the long term.

Meanwhile, repetitive transcranial magnetic stimulation (rTMS), a non-invasive and widely regarded safe treatment, is gaining traction for its applications in neurological and psychiatric conditions. Li H. et al. have shown that rTMS not only improved cognitive performance but also increased the T3 hormone levels among elderly post-stroke patients with low thyroid hormone levels. Furthermore, their findings revealed a positive correlation between increased T3 levels and improved cognitive function, suggesting that rTMS may serve as an effective rehabilitative intervention for post-stroke cognitive impairment.

These studies emphasize the potential of both pharmacological and advanced non-pharmacological therapies in addressing cognitive decline, paving the way for more comprehensive and targeted preventive strategies.

## 4 Biomarkers of dementia risk states: advances in detection and risk assessment

The biomarkers for dementia risk states span a wide array of categories, including behavioral, neurophysiological, neuroimaging, biological, and statistical approaches (Shah et al., 2023; Yamasaki and Ikeda, 2024). Seven studies also provided insights into the early detection and risk assessment of high-risk conditions associated with dementia.

Gao et al. investigated the risk factors for CF among older adults in nursing homes, applying both logistic regression and decision tree modeling to assess their predictive performance. Their findings revealed that while both methods delivered comparable accuracy, each offered unique strengths. By integrating these approaches, predictive precision can be further improved, providing valuable insights to inform clinical practice and guide policy development.

Bai et al. have suggested that integrating handgrip strength into the concept of MCR was shown to improve the predictions of dementia and all-cause mortality. The authors concluded that a modified MCR, incorporating handgrip strength, holds significant potential as an effective screening tool for detecting individuals at risk of dementia and mortality in national health examinations.

Yuan et al. further advanced predictive tools by identifying education, physical exercise, hyperlipidemia, osteoarthritis, depression, and Timed Up and Go test time as independent risk factors for MCR syndrome. They developed a nomogram model that demonstrated high accuracy, making it a valuable resource for early detection.

Ye et al. revealed a strong association between oral health-related quality of life and MCI in older adults, emphasizing its potential role in cognitive decline. The Geriatric Oral Health Assessment Index provides a practical tool for evaluating oral health in older adults, enabling the timely detection of poor oral conditions to help mitigate cognitive decline.

Tanaka et al. conducted a comprehensive review of studies on electroencephalography (EEG) markers for the early detection of dementia-related precursors, such as SCD and CF. The review emphasized advanced EEG techniques, including event-related potentials, quantitative EEG, microstate analysis, functional connectivity approaches, and the integration of artificial intelligence. Their findings suggested the potential of EEG as a non-invasive, cost-effective tool to identify individuals at risk, paving the way for timely interventions and personalized therapeutic strategies.

Frailty and increased serum neurofilament light chain (sNfL) levels are both closely associated with cognitive impairment. Yang et al. found a significant association between frailty and increased sNfL levels in a representative US population, with the estimated glomerular filtration rate partially mediating this relationship. These findings indicate that sNfL is a promising biomarker for frailty-related neuronal damage and emphasize the critical role of kidney function in this interplay.

Non-alcoholic fatty liver disease (NAFLD) has been associated with a heightened risk of dementia and cognitive decline. Wu et al. revealed a positive correlation between higher serum klotho levels and better cognitive performance in patients with NAFLD, suggesting that routine klotho testing can be used as a valuable tool for the early detection of cognitive decline in this population.

These studies collectively emphasize the importance of integrating diverse biomarkers and risk factors into innovative predictive frameworks, providing new opportunities for early detection and intervention in high-risk dementia states.

## 5 Unveiling research trends through bibliometric analysis

Bibliometric analysis is a systematic approach to studying scientific literature, along with identifying patterns, trends, and impacts within a field by applying quantitative methods to data collected and refined from relevant databases (Passas, 2024). This Research Topic encompasses two key bibliometric studies, each offering significant contributions to their respective fields.

Xiao et al. performed a bibliometric analysis to determine the key factors linking health behaviors to MCI. Their study emphasized five major research hotspots: exercise, diet, risk factors and preventive measures for dementia, cognitive decline-related biomarkers, and clinical trials.

Moreover, Wang et al. conducted a bibliometric analysis to investigate the research trends and key topics on social frailty among older adults. The key research hotspots in this field include social vulnerability, health, frailty, mortality, and older adults, while emerging trends emphasize dementia, Alzheimer's disease, population, and COVID-19 in the context of social frailty among older adults.

These findings provide valuable perspectives for researchers, aiding in the identification of critical contributions and shaping future investigations in this domain.

## 6 Conclusion

This Research Topic brings together pivotal studies on the risk factors for dementia-related high-risk conditions, innovative prevention and treatment strategies, emerging biomarkers, and bibliometric analyses. Collectively, these findings emphasize the critical importance of multidisciplinary approaches for early intervention and comprehensive management to mitigate cognitive decline. We believe the findings of these studies serve as a valuable resource, guiding clinicians in their practice and inspiring researchers in their pursuit of future advancements.
